# Coaching self-defense under COVID-19: challenges and solutions in the police and civilian domain

**DOI:** 10.1057/s41284-020-00269-9

**Published:** 2020-11-22

**Authors:** Swen Koerner, Mario S. Staller

**Affiliations:** 1 Department for Training Pedagogy and Martial Research, German Sport University Cologne, Am Sportplatz Müngersdorf 6, 50933 Cologne, Germany; 2 University of Applied Sciences for Police and Public Administration North-Rhine-Westphalia, Dennewartstraße 25-27, 52068 Aachen, Germany

**Keywords:** Self-defense training, Police training, COVID-19, Coaching, Expertise, Ecological dynamics

## Abstract

The spread of SARS-CoV-2 has led to a general shutdown of police and civilian self-defense training. While means of distance learning such as online teaching appear to be feasible for theory dominant subjects addressing cognitive resources of the learner, combat-related practices like self-defense trainings don´t seem to fit into the realm of virtual learning due to their bodily foundation. This is made clear by the collective perplexity of police and civilian coaches, gyms and organizations, on how to proceed with training during the lockdown in general, while approaches of distance learning (e.g., online learning) have rarely been considered. In the following article, we tackle the situation of police and civilian self-defense coaches in times of Corona. In a first step, contextual changes and challenges of coaching self-defense are identified through the lenses of a professional coaching model. In line with basic assumptions of ecological dynamics, *adaptability* seems to be the decisive resource for the coaching and training of self-defense in times of Corona. As an example for such an adaptation in training practice, a conceptual framework for distance-based self-defense training in the civilian and police domain is presented. This framework is adjusted to the respective requirements of physical distancing and adopted to novel security matters within the public sphere *caused by* the current regulations. In sum, the article attempts to provide ideas and orientation for police and civilian self-defense coaches as well as for their own development possibilities*.*

## Introduction

The corona pandemic triggered by SARS-CoV-2 poses major challenges to modern society worldwide. The development of COVID-19 is dynamic in nature and requires regionally and nationally adapted decisions depending on the current situation (Adam 2020). In their formal structure, political decisions related to the corona crisis correspond to a type of decision-making, for which Calabrese and Bobbitt coined the term "tragic choices" (Calabresi and Bobbitt 1978): The tragedy of choice is situated in the fact that positive effects in one regard are accompanied by negative outcomes in the other. While the political decision for a collectively binding lockdown in work and leisure slows down the spread of the virus, as it is clearly a current fact for Germany (RKI 2020), the associated measures build major challenges for numerous fields of modern society. Especially self-defense training in the civilian and police domain is affected by the restrictions on social interaction in a fundamental way (Andreucci 2020), since, for most of its practices and application contexts, direct physical contact is a key (Krabben et al. 2019).

The spread of SARS-CoV-2 in Germany has led to a general shutdown of self-defense training in civilian schools and gyms as well as in police organizations since mid of march 2020 (BR 2020). While means of distance learning such as online teaching appear to be feasible for theory dominant subjects addressing cognitive resources of the learner, practices like self-defense training does not seem to fit into the realm of virtual learning due to their bodily foundation. This has become obvious by the collective perplexity of individual and collective actors within the civilian and police domain, on how to proceed with training during the lockdown in general, while approaches of distance learning remain vague.

Even if the current easing of contact restrictions allows for a gradual return to training and interpersonal interaction at a distance of 1,5 to 2 m (DOSB ), it is still hard to predict when regular training as in the days before the COVID-19 pandemic will be possible. Similarly, a renewed wave of infection could result in a return to lockdown and thus a ban on direct training (Heiden and Buchholz 2020). However, in view of the health risks, a return to normal training appears to be more (2020) likely to be advisable in cautious steps.

In the following article, we tackle the situation of police and civilian self-defense coaches in times of corona. In a first step, (2) contextual changes (a) and challenges of coaching self-defense under contextual conditions of physical distancing (b) are identified through the lenses of a professional coaching model. According to basic assumptions of *ecological dynamics* (3), adaptability seems to be the decisive resource for professional coaching and training of self-defense in times of COVID-19. As an example for such an adaptation in training practice, (b) a conceptual framework for distance-based self-defense training within the police and civilian domain is presented and adjusted to the respective requirements of physical distancing and adopted to novel security matters within the public sphere *caused by* the current regulations. Overall, the article aims at providing ideas and orientation for police and civilian self-defense coaches as well as for possibilities for their own development.

## Contextual changes and challenges for self-defense coaches

### Coaching and contextual changes

**Fig. 1:**
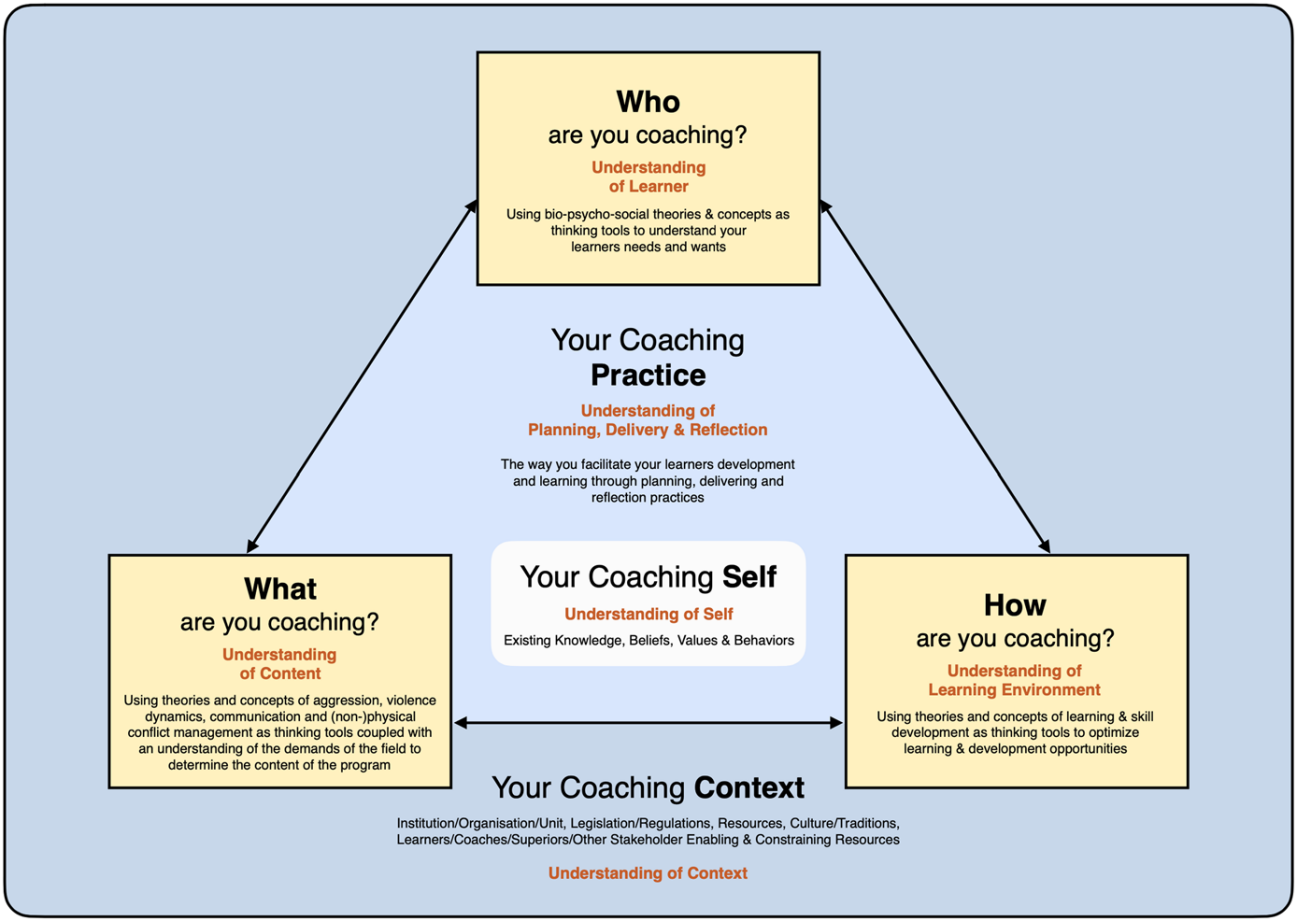
Professional coaching model (Staller 2020)

Coaching in general can be characterized as a complex decision-making process (Abraham and Collins 2011; Lyle 2018), posing high demands on the individual coach and coach education. The coaching model developed by Muir and colleagues (Muir et al. 2011; Till et al. 2019), which has recently been modified for combative contexts (Staller 2020), conceptualizes coaching according to six central dimensions that reflect the complexity and dynamics of the coaching process (see Fig. 1).

Accordingly, the central task of coaches is to orientate the planning and practice of training to the characteristics and requirements of the criterion environment of self-defense in the civilian and police domain (*what dimension*), to include the prerequisites and initial states of the learners (level of knowledge, motivation, developmental prerequisites, etc.) (*who dimension*), and to adjust the teaching method (*how dimension*). However, neither planning nor training itself resides in a vacuum, but are rather embedded in the personal characteristics of the coach (*self dimension*) as well as in the specific social and organizational (*context dimension*). The *practice dimension* includes the practice of planning, the training itself, and the reflection of one's own actions under the continuous influence of changing parameters.

Within this network, individual dimensions of the model are deeply interconnected: changes to individual parameters affect the system as a whole. For example, changes in the training environment (*context dimension*) can lead to changes in training content (*what dimension*) and delivery (*how dimension*), which may affect the actual motivation of learners (*who dimension*). The availability or necessity of a new training environment in turn has an effect on the design of weekly training classes (*practice dimension*), which can be designed differently in relation to the (un)conscious own values and the motivation of the trainer in connection with the altered situation (*self dimension*). While contextual changes in self-defense training usually show up in the area of material development (e.g., body protection, training pads) or in the resources available (training facilities, training partners), the current COVID-19 pandemic as a socio-cultural context variable is leading to changes in social context of self-defense training with a corresponding influence on coaching practice. Viewed from the context of training under normal conditions with direct physical contact (context phase 0), the COVID-19 pandemic has caused two different contextual conditions and phases so far.

#### Context phase 1—lockdown

The phase of lockdown is characterized by a standstill of self-defense training in police organizations, clubs, and gyms (Andreucci 2020). Contacts with other people beyond the own household are strongly limited by law. The life of the learners takes place mostly within their own home.

#### Context phase 2—cautious approach and risk minimization

The second phase is characterized by risk minimization of COVID-19 transmission and includes keeping a distance (min 1.5—2 m) between the persons present during training, giving preference to outdoor activities, reducing the size of training groups, and protecting members of risk groups (DOSB 2020).

Since the development of the pandemic can hardly be predicted at the present time (Heiden and Buchholz 2020), all contextual phases are in principle subject to change. For Germany, which is currently proceeding in phase 2, a transition to normality (phase 0) is just as conceivable as a relapse into phase 1 (Lockdown) in the event of a renewed wave of infection. In the following, we will focus on contexts 1 and 2, as we assume that civilian and police self-defense coaches face special challenges due to the novelty of both of these contexts (Andreucci 2020).

### Challenges

The lockdown phase (context phase 1), in which direct contact to people outside the own family is prohibited as well as the phase of training under distance conditions (context phase 2) is linked to challenges for coaches in self-defense-related professions.

For the *who dimension (1)*, motivational aspects of the learners as well as aspects of the coach-learner relationship are challenged, since the prescribed measures of physical distancing prevent the possibility for normal training and competitions and tend to result in self-defense learners retreating into the private sphere. In both cases, the question arises under which conditions, the social relationship between coaches, the individual learner, and learning groups can be maintained. The training has to be adapted (a) to the COVID-related regulations and (b) according to the needs and expectations of the learners, ranging from the wish for safety and health on the one hand, and the desire for social contact and continuation of the training on the other.

In the *what dimension (2)*, coaches are faced with the task of identifying, developing, and restructuring training content which a) is possible under the current restrictions and b) which make sense in terms of the respective criterion context. For self-defense training, the environment, where skills have to be applied, has been altered. Wearing of mouth–nose masks or the shift of life into the private sphere changes the characteristics and the dynamics of social conflicts civilians and police officers are facing. There is uncertainty about how the selected self-defense content in training will fit into the long-term development of the learners and what relevance it will have in future criterion contexts. Concerning the curricula that already exist and that have to be taught (e.g., in police recruits’ education), questions about restructuring, cutting down or changing content begin to rise.

In the *how dimension (3)*, coaches are faced with the question of how identified content can be designed in such a way that the learning environment meets the requirements of the criterion environment. Since interaction in most context of self-defense training is based on physical proximity and contact, fully representative training activities are not possible in either context (1 and 2). Especially in the domain of visual and kinaesthetic stimulation with interacting partners and environmental factors (e.g., confined spaces, in a car), appropriate solutions are required.

Within the *practice dimension (4)*, challenges of the what, how, and who dimension converge on the question of how martial arts training in times of corona as a whole can be planned and executed. For self-defense training, there is no reference experience in comparable contexts. Due to the novelty of the situation, a great deal of time must be expected when planning and organizing the training and linking it to medium- and long-term training objectives. For the teaching practice itself, the question arises as to how meaningful tasks can be designed, how interaction can be arranged and how feedback can be given for the respective practice. Overall, the future relevance of the efforts made within this domain remains uncertain.

For the dimension of the *coaching self* (5), the current restrictions may irritate existing beliefs, values, and attitudes towards the training process. Thoughts such as "this can't be done" or "that's not the way training has to be done" are possible here. The challenge is to identify and work on one's own assumptions that guide one's actions in relation to training and to open up to new, previously unknown ways of thinking and acting.

The list of challenges is admittedly remarkable: physical proximity between learners and coaches is limited, social relations to the athletes have to be maintained, relevant training content has to be identified, and familiar ways of interaction and delivery have to be adapted. Skill development is difficult to assess, the organizational effort is much greater than before COVID-19 pandemic and overall, there is a lack of reference experiences. Hence, it is quite understandable when police and civilian self-defense coaches initially react irritated. However, as it is known for the learning processes in general (Dewey 1910), the phase of irritation can merge into learning, as soon as each of these problems is viewed as currently given constraint conditions and thus with their inherent potential for development possibilities. Not quite incidentally, this exactly is the mindset self-defense coaches expect of their learners: To overcome the shock of an unexpected situation and turn into adaptive, creative *problem-solvers* (Staller and Abraham 2016).

## A conceptual framework for self-defense coaches

The plea for adaptivity of self-defense coaches can also be substantiated considering key ideas of *ecological dynamics* (Roberts et al. 2019). Ecological dynamics supposes (a) a mutuality of individual and environment, meaning that individuals perceive the environment and create the environment at the same time (Gibson 1979), and (b) the paramount role of individual, task, and environmental *constraints* (Newell 1986), delivering individuals opportunities for action and allowing them to attune to information, which guide their behavior (Koerner and Staller 2020; Renshaw et al. 2010; Renshaw and Chow 2019). The peculiarity of constraints in view of ecological dynamics lies in the fact that they constitute both limitations and possibilities of behavior at the same time (Torrents et al. 2020).

### Key constraint—the coaching self

By situating the individual self-defense coach not in the position of an external observer, but as a relevant player within the ecological approach (Orth et al. 2018), contextual changes caused by SARS-CoV-2 can be seen as environmental constraints resulting in challenging tasks to which the coach behaves in several possible ways, ideally taking them as opportunities for action. As mentioned before, how coaches attune to the specific unfamiliar situation depends on the personal mindset, which is acting as an individual constraint and affordance in the light of ecological dynamics. Ecological dynamics does not only allow for a theory-based description of the demanding situation police and civilian self-defense coaches (*self dimension*) are currently confronted with. Providing the basis for a "principled approach to skill learning across all sports and in all pedagogical settings" (Renshaw and Chow 2019, p. 104), *ecological dynamics* offers concrete orientations for the design of self-defense training and thus for a constructive approach to the requirements discussed.

### Constraining the what dimension

Under the conditions of lockdown (context phase 1) and distance regulation (context phase 2), both the criterion and the training environment of police and civilian self-defense have been changed. Self-defense practices and police use of force refer to interpersonal threat and conflict dynamics in the public sphere, which may be influenced and changed by the measures of contact restriction. For instance, the following new scenarios caused by SARS-CoV-2 and corresponding regularities are conceivable: (a) spatial isolation could lead to an increase in incidents of domestic violence and less available support services (Usher et al. 2020). Furthermore, (b) an overall increased tension in the population due to the novel situation, (c) the removal of a mouth and nose protector at the corresponding obligation to wear it, (d) navigating between large crowds with a minimum distance, (e) the falling below the minimum distance, or (f) cases of aggression aiming at transmission of SARS-CoV-2 bears an enormous potential for conflict between people in public spaces. The wearing of a mouth and nose protector alone changes the situational parameters and quality of conflict dynamics. For example, facial expressions, gestures, and acoustics of the interacting persons which are covered by the "mask" or which can only be perceived to a limited extent imply the possibility of not being recognized or not being recognized in time or of being misinterpreted, which makes it difficult, among other things, to send and attune to de-escalating signals (e.g., via a smile). In addition, mouth–nose protection restricts the supply of air, which can increase the physiological arousal of conflict partners and lead to an increasing restriction of cognitive and physical capabilities.

The identification of these and other contextual and situational parameters of civil conflicts provides specific clues for the *training environment* of police and civilian self-defense practices to expand and differentiate the scope of what to be taught, e.g., coping with specific corona scenarios under the described restrictions.

For the training environment of civilian self-defense and police training, current restrictions in interpersonal contact result in the following opportunities for the selection of content:

1. Focus on basic and complementary skills that promote the development of key action capabilities in the long term, including the development of physical performance (e.g., general and specific fitness training), basic motor skills, as well as specific skills such as training of explosive attacking actions (Staller et al. 2018, 2020a). The isolated training of defensive actions also offers opportunities for functional development of learner`s competence experience.
2. Expansion of declarative knowledge structures through lectures, discussions, and video analyses, which can lead to a deeper understanding with regard to the subject matter.

Once possible contents of civil and police self-defense training under conditions of corona have been identified, the question for trainers is raised, how these topics can be implemented in training in a representative manner so that the learner's activities within the training environment meet the requirements of the criterion environment.

### Constraining the how dimension

The contextual conditions of training at a distance are linked to challenges for coaches in terms of task design. For example, in most types of self-defense training, direct physical contact with changing training partners is required. Fighting generally can be conceptualized as a mode of physical communication, established through the dense interaction of bodies (Körner et al. 2019; Krabben et al. 2019; Staller and Körner 2019). It is central to skill development in self-defense and police training that coaches design training activities in a representative manner (Pinder et al. 2011; Staller et al. 2017). Exercises and tasks are representative if they resemble key requirements set in the application context (functionality) and thus enable the trainee to behave as he/she should behave in the criterion environment (action fidelity, Pinder et al. 2011). This includes the focus on behavior-specific information in a) physical (e.g., dealing with speed and force), b) perceptual-cognitive (e.g., dealing with surprise), and c) affective (dealing with emotions) terms as well as the exploration of adequate coping strategies (Broadbent et al. 2015; Headrick et al. 2015; Komar et al. 2019; Maloney et al. 2018).

**Fig. 2:**
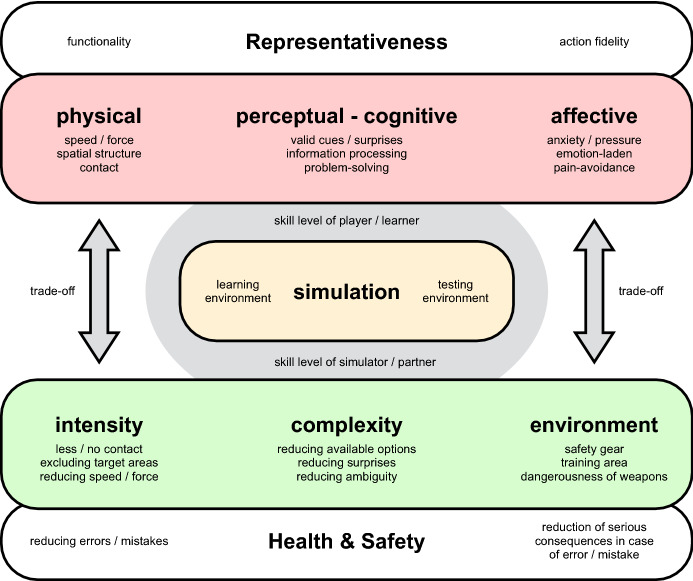
The trade-off model of simulation design (Staller et al. 2017)

Splitting up the representativeness of a learning task (Fig. 2) allows the self-defense coach to "play" in a way that is similar to playing at a mixing desk. In the totality of the simulations performed in a training program, it can thus be ensured that central elements of the criterion environment must be played in step by step, varied gradually, and treated by learners (Körner and Staller 2017). While under "normal" training conditions, martial arts training aims to ensure a high overall representativeness of tasks, current coaching contexts require an increased splitting up of representativeness and ensuring that a high representativeness is maintained in the sum of individual tasks and exercises. This approach is not new in self-defense training, since a comprehensive overall representativeness cannot be guaranteed due to the risks to the health and safety of the trainee (Staller et al. 2017).

Contemporary context and regulations of physical distancing require police and civilian self-defense coaches to apply and develop task designs, for example, show a high degree of representativeness in the perceptual-cognitive area, while at the same time being less representative in the physical component. For instance, the following options are feasible:

1. Interaction of functional optical and acoustic stimuli allowing for action fidelity while maintaining distance, e.g., simulation of attacking actions in the area of kicking or striking techniques by ecological valid triggering stimuli (e.g., weight shift by the attacker/simulator), presenting opportunities for defense actions which have to be perceived and executed by the player (defender).
2. Interaction of functional kinaesthetic stimuli and valid responses while maintaining distance. Here, creative coaching methods must be explored to ensure interactions while maintaining the minimum distance. Initial ideas (practiced by the authors) include the use of kinaesthetic "bridges" such as "pool noodles" (of 1.5 to 2 m length) for attack variations on which defense actions can be performed or the use of ropes to ensure tension–compression movements in the interaction.

### Constraining the practice dimension

The identified contents and design ideas presented finally lead into the *practical dimension*. How can those ideas and plans be implemented in practice, for example in the phase of lockdown (context 1), where interaction with martial arts learners is only possible as interaction among absentees via modern media. What forms of delivery and pedagogical approaches are available here? To which amount different approaches are appropriate and how can they technically be realized? Where is the place for linear pedagogy, advocating coach-centered demonstration and explanation of ideal solutions and imitation by the learners? Where is a place for non-linear pedagogy (Chow et al. 2016; Körner and Staller 2018; Koerner et al. 2020), offering learners the opportunity to make individual decisions, exploring individual solutions-based interactively provided tasks and cues? The latter, for instance, could be realized within a synchronic online training (e.g., via Zoom, Webex).

For example, if in simulation of a punch attack by the coach, visually and acoustically mediated by the camera, the learners themselves are left to decide what (what-decision) to do and how (how-decision). In this case, task design as well as the delivery affords learners' exploration and exploitation of functional movement variability. Opportunities for learners defense and counter actions are created through the deliberate manipulation of constraints and are thus set by the task (not to get hit), the environment (learners may have to train in private rooms, to put a chair between themselves and the screen, etc.) and through individual constraints, e.g., by wearing a rucksack while performing defense and follow-up actions. But how can individual real-time feedback be delivered and technically be managed on screen? Is there a use for all of this in future? How does learning this way contribute to long-time learning and performance goals? There is no doubt that many open questions are linked to the coaching practice of police and civilian self-defense training in times of Corona—answers to these questions can only be found by doing it, by teaching (Table 1).

**Table 1:** Impact of contextual changes (context phase 2)

Dimension	Contextual changes (context 2)	Challenges	Possibilities	Limits
What	Changing the application environment New conflict scenarios and situational dynamics Changes of the learning environment Training is again allowed at a distance < 1.5 m from the partner in close proximity (compared to phase 1)	Identification (and development) of training content whose training is possible and fits into the long-term development of the learners regarding needed competencies for performance within the criterion environment	Application environment Expansion of possible training contents Learning environment Focus on complementary skills (fitness, technique, situational awareness etc.) Expansion of declarative knowledge (video analysis of application situations, presentations)	Certain training contents prohibited (e.g., ground fighting, choking techniques)
How	No contact allowed Outdoor training Smaller groups	Design of representative learning environments	Compared to phase 1 More partners for (visual/acoustic) stimuli and interactions Possibility of interaction involving tension/pressure (kinaesthetic stimulation)	completely representative training activities not possible Kinaesthetic stimulation only partially representative Interaction of visual information and actions only partially representative
Who	Contact is not allowed Seeing others is allowed "Desire" for togetherness	Identification and consideration of current wishes, needs, and expectations of the learners	Getting to know the learners better Focus on relation between athlete and coach: growing together in times of crisis	Retreat of learners/trainees into the private area possible Motivational problems due to lack normality/usual training
Self	New situation as danger/insecurity" Confrontation of own beliefs about training; danger of "I can't"/"this is not feasible""	Identification of own attitudes, values, and resources that guide actions with regard to training	Recognition of own assumptions that guide actions Training of creativity, adaptivity, and flexibility in training design	Future relevance unclear/uncertain
Practice	Planning: no reference experiences with training in comparable contexts; problems in future orientation (periodization) Time-consuming implementation under general conditions and requirements Delivery: No direct proximity to learners; different "feeling" of the training; organizational effort Reflection: Feedback about "what works" not directly visible (in the application environment)	Development/adaptation/strengthening of existing planning, implementation, and reflection structures More theory-driven and experimental instead of experience-based More organizational effort Systematic evaluation possible	Trying out new training interventions, ways of delivery, supporting material/media/technology Discussion of the theoretical justification for the use of specific forms of training Opening up new possibilities for reflection and evaluation on the effectiveness of training activities	Future relevance unclear/uncertain
Context	Rules and regulations on contact restrictions between persons	Creation of contextual conditions that allow self-defense training with individuals under current regulations	Training groups that train under quarantine conditions Training with persons from a household	Not possible/allowed for all learners/trainees at all levels

By becoming active in times of Corona and offering training, by entering unfamiliar terrain, e.g., in the field of technical forms of communication (e.g., via zoom), by testing online-based distance learning, by adapting approaches to delivery, and by designing novel tasks, self-defense coaches embody a sense of community and social relatedness—and thus move into the core of the learners needs and expectations (*who dimension*). As motivation research has repeatedly argued for the positive influence of measures to promote and stabilize social relatedness on motivation (Mageau and Vallerand 2003; Rigby and Ryan 2018), it can be assumed that especially in times of obligate physical distance, learners appreciate the willingness and initiative of their coaches to find alternative solutions of contact and interaction. Also, and precisely because the situation obviously forces police and civilian self-defense coaches to leave their comfort zone, the situation automatically leads to the development and opening up of new expertise in the field of media and pedagogy and therefore to possibilities for professional development.

## Conclusion

Police and self-defense coaching are challenged by the current COVID-19 pandemic and the associated measures of physical distancing. Due to a lack of experience, simple answers and solutions are not to be expected. At first glance, it may seem strange for police and civilian coaches to get involved in the possibilities of self-defense training under conditions of current contact restrictions; after all, self-defense normally includes proximity and direct physical contact. In this article, we argue that we should not leave it at the defensive reflex, but rather see the crisis as a potential for the adaptation of professional coaching practice and thus make it the starting point for our own development opportunities. As such, the COVID-19 pandemic provides an opportunity to redevelop coaching expertise (Turner et al. 2012; Staller and Körner 2020b) and by this developing, the ability enabling them to decisively respond to new expertise demands that arise as a result of changes in their expertise territories: “flexpertise” (Frie et al. 2018). This is preceded by the willingness to do exactly what police and civilian self-defense coaches expect of their learners to do on a regular basis: overcoming the shock of an unexpected situation and becoming adaptive, creative problem-solvers.
